# Mercury and cyanide exposure and safety practices in Sudanese artisanal gold mining communities, 2022

**DOI:** 10.1539/eohp.2025-0035

**Published:** 2025-12-24

**Authors:** Shafee S. Almahi, Mohammednour Mukhtar Mohammednour Ali, Mohammed O. Adam, Mohammedahmed M. Osman

**Affiliations:** 1 Faculty of Medicine, University of Khartoum, Khartoum, Sudan

**Keywords:** ASGM, cyanide, exposure, hazards, mercury, PPE use

## Abstract

**Background:**

Artisanal and small-scale gold mining (ASGM) involves informal, small-scale investments in gold mining using low technology. It accounts for 39% of annual mercury emissions, in addition to cyanide emissions. Mercury and cyanide have toxic effects on health and environment, especially from direct exposure. Protective measures and knowledge are crucial for controlling these hazards. We aimed in this study to assess health impacts and knowledge about mercury and cyanide, as well as attitudes regarding personal protective equipment (PPE) among gold miners directly exposed to these toxicants.

**Methods:**

This community-based cross-sectional study was conducted at a mining site in River-Nile State using a self-structured questionnaire. The study included 269 participants, and the data were analyzed using SPSS. Descriptive statistics were employed to summarize and present the data.

**Results:**

The mean age was 31 years. Elemental mercury was the mostly used chemical (98.5%), exposure forms included hand and foot handling (64.5%) and vapor (35.5%). Cyanide was used by 7.8%, exposure forms included vapor (60%) and hand handling (50%). The most reported complaints were persistent headache (32%), numbness and tingling (10%), itching (9.5%), and tremors (7%). PPE was not used by 52.5% of participants. The average overall knowledge score was 8.7/17. PPE use was associated with higher overall knowledge and lower prevalence of headache and skin rash (p<0.05). Long daily working hours were associated with headache, while prolonged work duration was associated with numbness (p<0.05).

**Conclusion:**

The majority of participants did not use PPE and had low average overall knowledge about mercury and cyanide toxicity.

## Introduction

According to the international Minamata Convention on mercury control held in Japan in October 2013, artisanal and small-scale gold mining (ASGM) is defined as: gold mining individuals or small businesses with limited capital investment and production. ASGM is accounted for the largest anthropogenic mercury emissions for more 39% of the world’s annual mercury emissions^1,2)^ and accounted for 20% of the annual global gold production. Over 40 million people in over 70 countries are involved in ASGM^3)^, and this count is keeping on rising in recent years, especially with the downturn in the economy following the coronavirus pandemic; in fact, ASGM becomes a cornerstone in rural economic development^4)^.

In ASGM, elemental mercury extracts gold from ore as an amalgam, then the amalgam is isolated by hand and heated to distill mercury and separate gold^1)^. This technique is cheap, simple, and quick^5)^, but not so effective to maximize economic gain due to lack of technical expertise and knowledge of the mining sites’ geography, and mineralogy^6)^; moreover ASGM is more likely to detriment health, society, and the environment.

Mercury exists as organic, inorganic, and elemental forms and comes into contact to us through skin and eye (gold mining), inhalation (gold mining and agricultural fungicides), and ingestion (dental amalgams, contaminated fish and grains). Skin and eye contact causes skin rash and eye irritation with minimal systemic toxicity^7)^. Elemental mercury vapor as in ASGM is converted into inorganic mercury inside the body; 90% of ingested organic mercury is readily absorbed, while 10% of ingested elemental form is poorly absorbed^8)^. Once inside the body, mercury resides in the kidneys and brain and inactivates enzymes, leading to wide range of neurological and renal manifestations (Minamata disease)^5,8,9,10,11)^.

Cyanide (the rapidly killing asphyxiant gas) is commonly used in ASGM in form of cyanide salt to stabilize gold in solution^12,13)^. In addition to its environmental hazard, miners’ knowledge about its risk and antidote is crucial. Cyanide is also a culprit in kidney disease and can form mercury-cyanide complexes, rendering it more toxic than inorganic mercury^14)^.

Different ways exist to control mercury hazard: elimination, substitution, engineering controls, administrative controls, and personal protective equipment (PPE). PPE use is deemed the least effective strategy, despite the fact that it is cheap, simple, and widely recommended^15)^. Workers tend to have poor PPE compliance^8)^. The ASGMs’ lack of formal education and training in mercury toxicity and management procedures are to blame for the high non-compliance with PPE use^16,17)^.

Sudan ranks third in Africa and 15th globally in gold production, with ASGM accounting for 85% of the country’s gold output and engaging approximately one million miners^17,18)^. The environmental and health hazards associated with mercury and cyanide use in ASGM are substantial and poorly understood, particularly in regions like River Nile State, where limited studies have been conducted. Despite the serious health risks, PPE use is minimal, largely due to a lack of formal education and training among miners.

This study seeks to address this knowledge gap by evaluating miners’ exposure to mercury and cyanide, their awareness of associated risks, and their compliance with safety protocols in River Nile State. Given ASGM’s critical role in Sudan’s economy and its profound health implications, this research aims to contribute valuable insights for health authorities to establish more effective regulations and to design educational interventions tailored to mining communities. Ultimately, these findings will support the development of safety protocols that protect miners and reduce the environmental impact of ASGM in Sudan.

## Methods

### Study design and setting

The study was community-based cross-sectional study in a mining area of “Altawahin” in the district of Alabediah, River Nile State, Sudan. Data was collected from November 14–30, 2022.

### Sampling

We used a convenience sampling method and calculated the sample size using the formula

N=z^2^ p (1−p) / e^2^

Where Z is the z score (1.96)

e is the margin of error (0.05)

p is the population proportion (0.5)

Mine workers in the study area with current or history of usage of elemental mercury, cyanide, or both were included. Those who were unwilling to participate in the study were excluded.

### Data collection and analysis

We collected the data using a self-report questionnaire (eMaterial 1) composed of four sections; socio-demographic data, exposure, hazards, PPE attitude, and knowledge about mercury and cyanide. We distributed a paper-based version and an electronic version of the questionnaire.

Knowledge about mercury and cyanide was assessed using 17 questions with three options of: yes, no, and I don’t know. Each question has one correct answer with a score of 1, while incorrect answers have a score of zero. The total knowledge score for each participant ranged between zero and 17.

We analyzed the data using IBM SPSS v.26 (IBM Corp, Armonk, NY, USA). We used descriptive statistics and non-parametric tests of Chi-square of independence, Mann-Whitney U, and Kruskall-Wallis tests to analyze the data, with p-values considered significant at 0.05. Tables, figures, and charts were used to present the results.

### Ethical consideration

Ethical approval was attained from the Department of Community Medicine, University of Khartoum. Informed Consent was taken from each participant.

## Results

### Sociodemographic

The questionnaire was completed by 269 miners. The participants in this study had a mean age of 31.5 years, and about two-thirds of them ranged from 22 to 40 years. There was only one female participant in this study (0.4%). Most of the participants completed their secondary school learning; university education was more common than primary school only, one participant had postgraduate education, and none of the participants were illiterate.

Elemental mercury was used far more often than cyanide in amalgam, and hand handling outweighed vapor exposure for elemental mercury, in contrast to cyanide, in which vapor exposure slightly predominated (Table 1). Almost half of the participants work in gold mining sites located less than 5 kilometers from the nearest villages.

**Table 1.** Sociodemographic data, type of chemicals used and mode of exposure (N=269)

**Age in years**	Mean **(31.5)**	Min.	17
	Standard deviation **(9.1)**	Max.	70
		**Frequency**	**Percentage %**
**Gender**	Male	268	99.6%
	Female	1	0.4%
**Education**	Primary	50	18.6%
	Secondary	156	58.0%
	University	62	23.0%
	Postgraduate education	1	0.4%
**Type of chemical used**	Elemental Mercury	248	92.2%
	Cyanide	4	1.5%
	Both	17	6.3%
**Elemental mercury exposure**	Hands	189	44.7%
	Vapor	150	35.5%
	Foot	84	19.9%
**Cyanide exposure**	Hands	8	40%
	Vapor	10	50%
	Both	2	10%
**Distance**	≤200 meters	20	7.5%
	201–1000 meters	14	5.2%
	>1–3 kilometer	37	13.8%
	>3–5 kilometer	66	24.5%
	>5 kilometer	132	49.1%

### Personal protective measures

**Fig. 1.**
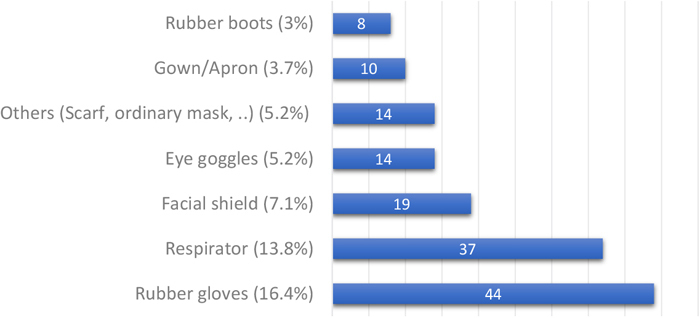
Frequency and percentage of PPE use (N=269)

More than half of participants did not use PPE, but the majority of them reported washing hands and rinsing after work (Table 2). Rubber gloves and respirators were the most commonly used PPE (Figure 1). A chi-square test of independence was conducted to examine the association between the use of PPE and educational level and revealed no statistically significant association (p=0.37). This indicates that the observed patterns of PPE use did not significantly differ across different educational levels.

**Table 2.** Frequency of different protective measures during work (N=269)

Protective measures	At least once N (%)	Never N (%)
Use of PPE during work	116 (47.5%)	128 (52.5%)
Washing the PPE after work	115 (45.1%)	140 (54.9%)
Washing your hands regularly after work	258 (95.9%)	11 (4.1%)
Rinsing your body regularly after work	258 (95.9%)	11 (4.1%)

PPE, personal protective equipment.

### Knowledge score

The mean overall knowledge score (out of 17) was 8.7 (standard deviation, 4.75), with minimum and maximum scores of 0 and 16, respectively. Counterintuitively, the Kruskall-Wallis test indicated that higher education was associated with lower mean rank of overall knowledge, but this was not statistically significant (p=0.146) (Table 3). The Mann-Whitney U test showed that use of PPE significantly reflected higher overall knowledge (p=0.004). The Kruskall-Wallis test indicated that participants working in mining for more than 3 years had significantly higher rank of overall knowledge (p-value<0.05) (Table 3).

**Table 3.** Association between overall knowledge with educational level, use of PPE, and work duration (N=269)

		Test used	N	Mean rank of overall knowledge (Mean 8.7; standard deviation, 4.7)	p-value
**Education**	Primary	Kruskall Wallis	50	147.8	0.146
	Secondary		156	136.9	
	University or higher		63	119.9	
**PPE use**	No use of PPE	Mann-Whitney U	177	125.1	0.004*
	Used at least 1 PPE		92	153.9	
**Working duration**	Less than 6 months	Kruskall Wallis	53	137.4	
	Between 6 months and 1 year		83	132.4	
	Between 1 year and 3 years		52	82.5	
	More than 3 years		**81**	**169.6**	0.001*

PPE, personal protective equipment.

* Statistically significant at 0.05

### Complaints reported

**Fig. 2.**
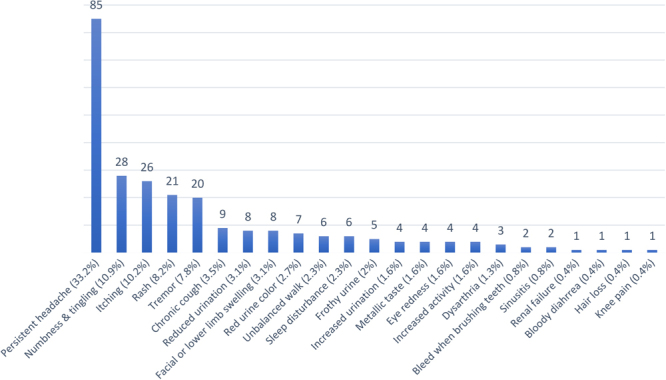
Frequency and percentage of complaints among participants (N=269)

Headache was the most commonly reported complaint, with approximately one third of the participants reporting headache; numbness/tingling sensation was the second-most common complaint (Figure 2).

The Chi-square test for independence showed that use of PPE was significantly associated with lower prevalence of headache and skin rash, but with more prevalence of hand tremors and numbness and tingling sensation (p<0.05) (Table 4).

**Table 4.** Chi-square tests for association between frequently reported symptoms among all participants with use of PPE, daily working hours, and work duration (N=269)

	Use of PPE			Daily work hours			Work duration		
	Never	At least once	P‑value	Less than or equal to 3 hours	More than 3 hours	P‑value	Less than or equal to 6 months	More than 6 months	P‑value
Hands tremor	7	13	0.003*	6	14	0.888	7	13	0.074
Headache	64	21	0.026*	13	72	0.001*	17	68	0.93
Numbness and tingling	12	16	0.007*	8	20	0.995	11	17	0.006*
Skin rash	18	3	0.045*	3	18	0.130	3	18	0.516
Itching	16	10	0.63	5	21	0.265	6	20	0.649

PPE, personal protective equipment.

* Statistically significant at 0.05

**Table 5.** Chi-square tests for association between use of different types of personal protective equipment and frequently reported symptoms

	Respirator		Facial shield		Eye Goggle		Gown (full sleeve-apron)		Rubber/ Nitrile gloves		Rubber boots	
	Yes	No	Yes	No	Yes	No	Yes	No	Yes	No	Yes	No
Hand tremors	7 (19%)	13 (6%)	1 (5%)	19 (8%)	2 (14%)	18 (7%)	1 (10%)	19 (7%)	6 (14%)	14 (6%)	1 (13%)	19 (7%)
	P*=0.011**		P*=1.000		P*=0.279		P*=0.545		P*=0.111		P*=0.466	
Headache	3 (8%)	82 (35%)	9 (47%)	76 (30%)	4 (29%)	81 (32%)	3 (30%)	82 (32%)	7 (16%)	78 (35%)	4 (50%)	81 (31%)
	P=0.001**		P=0.132		P*=1.000		P*=1.000		P=0.013**		P*=0.267	
Numbness and tinging	6 (16%)	22 (10%)	3 (16%)	25 (10%)	4 (29%)	24 (9%)	2 (20%)	26 (10%)	**7** **(16%)**	**21** **(9%)**	**1** **(13%)**	**27** **(10%)**
	P*=0.243		P*=0.430		P*=0.045**		P*=0.279		P*=0.186		P*=0.590	
Skin rash	1 (3%)	20 (9%)	1 (5%)	20 (8%)	0 (0%)	21 (8%)	0 (0%)	21 (8%)	1 (2%)	20 (9%)	0 (0%)	21 (8%)
	P*=0.327		P*=1.000		P*=0.611		P*=1.000		P*=0.216		P*=1.000	
**Itching**	5 (14%)	21 (9%)	0 (0%)	26 (10%)	0 (0%)	26 (10%)	0 (0%)	26 (10%)	5 (11%)	21 (9%)	0 (0%)	26 (10%)
	P*=0.374		P*=0.232		P*=0.374		P*=0.605		P*=0.780		P*=1.000	

P* =P-value for Fisher exact test is reported as at least 25% of cells had expected count less than 5.

** =P-value is significant at 0.05 level

Percentages reported are within columns (i.e % within PPE used)

Another chi-square test indicated that increasing working hours and work duration were associated with higher prevalence of the five most commonly reported symptoms; however, most of these findings were statistically insignificant (p>0.05) (Table 4).

Significant relationships were observed between several commonly reported symptoms and different types of PPE. Respirator use was associated with a higher occurrence of hand tremors (p=0.011) and a lower occurrence of headache (p=0.001). Eye goggle use was significantly linked to a higher report of numbness and tingling (p=0.045), while rubber/nitrile glove use showed a significant association with increased headache (p=0.013).

## Discussion

To our knowledge this is the first study to include only gold miners who have direct contact with elemental mercury and/or cyanide.

### Toxic mercury effects reported by the miners

In our study neurological effects were the most common reported by ASGM workers, represented by headache (n=85), followed by numbness and tingling (n=28), and lastly tremors (n=20); this is similar to some extent with the comprehensive study done by Gibb et al.^5)^, which concluded that neurological effects were the commonest, represented by tremor, ataxia, memory problems, and vision disorder. Surprisingly, another study in Sudan by Etaib et al.^17)^ reported headache by 57.1% of the workers, followed by neuropsychiatric problems. This high prevalence of headache in these two Sudanese studies (the present study and Etaib et al.) may be due to other occupational confounders, like long working hours and low water intake. For comparison, in two Ghanaian studies by Afrifa et al. and Mensah et al.^8,19)^, fatigue, skin rashes, red eyes, metallic taste, and numbness were the most commonly reported symptoms among Ghanaian small-scale gold miners who are exposed to mercury. As noted by Mensah et al.^19)^, numbness was significantly associated with mercury exposure (urine level). We found that numbness was significantly associated with work duration. Furthermore, skin rash was not significantly associated with mercury exposure in a study conducted by Mensah et al.^19)^; the present study found no association with work duration. Interestingly, however, in the current study, the low incidence of skin rash — reported exclusively among workers exposed to mercury alone or to both mercury and cyanide, regardless of the route of exposure — was significantly associated with the use of PPE.

### PPE usage and miner’s knowledge about Mercury hazards

More than half of participants reported no PPE use at all. This pattern of non-compliance to PPE seems to be shared by most ASGM communities worldwide, as Rojas et al.^15)^ found around 55.5% of gold miners have complete non-compliance to PPE in Venezuela, and Afrifa et al.^8)^ reported that around 86.55% of the gold miners in Ghana who participated in their study did not use PPE as recommended. In particular, it was revealed that 89.8% of people did not wear nasal masks to protect themselves from vaporized mercury in the study done by Afrifa et al.^8)^. These results were in line with previous studies that found gold miners used PPE infrequently, as noted by Mensah et al.^19)^. Spiegel et al.^16)^ supported the fact that miners and millers in a Tanzanian ASGM community were directly exposed to mercury during amalgamations as well as burning without retorts, which is a device that captures mercury during amalgam burning. In the current study, PPE use was associated with overall knowledge score (p=0.004) but was not significantly associated with the educational level (p=0.37). This may be due to variability in the availability or affordability of the PPE. Regarding the type of PPE used, rubber gloves were the most common, used by 16.4% of miners, and rubber boots were the least used, by only 3% of miners. This is in contrast to the study by Mensah et al.^19)^, which found that leather boots were the most common (9.6%) and rubber gloves were used by only 6.4% of miners. Apron usage was low in the present study and in the study by Mensah et al. (3.7% and 2%, respectively).

The mean of overall knowledge of the miners about mercury nature and its effects in the current was 8.7/17; that is, miners only knew about 50% of the hazards of mercury. Similarly, Mensah et al. found that 65.6% of miners did not know about mercury hazards. In the present study, 58% of participants reached secondary school; this is in contrast with Mensah et al., where about 60.9% of the participants stopped at primary school. We did not find overall knowledge to be significantly associated with the level of education. This low level of education and knowledge seems to be characteristic in these communities, as these individuals are mostly directed by economical aspects — searching for better status and an alternative that is rapid and easier than farming and may be an alternative to schooling^20)^. It is also worth noting that some miners in ASGM were seen handling metallic mercury with their bare hands without wearing gloves.

Overall, ASGM communities are very inconsistent: many of them are working illegally and highly mobile, not settling in one mining site; in addition, there are no available registered or authorized numbers of ASGM workers worldwide.

Because of the cross-sectional design, we could not evaluate previous exposures and short-term effects. Symptoms and signs were self-reported, which is subjective. In spite of these limitations, the data showed that mercury inebriation in ASGM workers is a significant, neglected worldwide health problem.

### PPE use and reported symptoms

The statistically significant association between respirator use and fewer headaches may reflect a real protective effect, possibly due to reduced exposure to mercury vapor, which has known neurotoxicity. Other associations — like glove use and headaches or goggle use and tingling — are less convincing and may be false positives due to small sample sizes, multiple testing, and marginally positive p-values. Larger studies with adjusted analyses are needed to confirm these findings.

## Conclusion

This study revealed that a considerable group of our community without university-level education engage in ASGM to offer a good socioeconomic status. Many ASGM workers have direct contact with mercury and cyanide, without even a barrier. The majority of the miners did not use PPE, but the majority reported washing hands and rinsing their bodies after working. Many of the complaints reported by the workers can be attributed to the toxic effects of mercury. These included persistent headache, numbness and tingling, hand tremor, itching, and skin rash. The average overall knowledge about mercury and its effects is about 50%. Future studies, educational programs, and workshops should be established in mine areas, and mining should be far away from residential areas. PPE should also be available and affordable, and alternative technologies should be implemented to reduce exposure to mercury emissions.

## Supplementary Material

Supplementary eMaterial 1

## Data Availability

The datasets used and/or analyzed during the current study are available from the corresponding author on reasonable request.
