# Supplementary material for: Mercury and cyanide exposure and safety practices in Sudanese artisanal gold mining communities, 2022
Source: Environ Occup Health Pract. 2025 Dec 24;8(1):2025-0035. doi: 10.1539/eohp.2025-0035 (PMC13012897; doi:10.1539/eohp.2025-0035)
Supplement: Supplementary file 1 — Supplementary eMaterial 1 [file eohp-8-2025-0035-s001.pdf]

eMaterial 1. Questionnaire

<u>Section (1): Sociodemographic:</u>

1) Age

2) Sex

- Male

- Female

3) Educational level

- Primary school

- Secondary school

- University or postgraduate education

<u>Section (2): Mercury and cyanide exposure:</u>

1) Do you use mercury in work?

- Yes

- No

2) How are you exposed to it?

- Handling.

- Foot.

- Exposure to its vapor

3) Do you use Cyanide salts in work?

- Yes

- No

4) How are you exposed to it?

- Handling.

- Exposure to gas

5) What is your daily working hours

0-1 hours

2-3 hours

3-4 hours

4-5 hours

> 5 hours

6) What is your working period?

- month - 3 months

- more than 3 - 6 months

- more than 6 months - 1year

- more than 1 year to 3 years

- more than 3 years

<u>Section (3): Complaints reported by the participants:</u>

Question: Have you felt or sense any of the following symptoms during or after daily work:

(Note: This does not include symptoms that were or are continuing before the period of engaging in this work)

1- Tremors and tremors in the hands or body (or noticed by others)

2- Chronic headache/headache

3- Unbalance and staggering in walking

4- Dysarthria

5- Excitability

6- Sleeping problems

7- Numbness and tingling

8- Itchy skin

9- skin rash

10- Redness in the eyes (others noticed)

11- Chronic cough

12- Inflammation of the gums(gingivitis): bleed when brushing teeth

13- Diarrhea containing blood

14- A metallic taste/taste in the mouth

15- Thyroid problems

16- Anemia

17- Oliguria

18- Polyuria

19- Frothy urine

20- Lower limb or facial swelling

21- Red urine

Any other symptom not mentioned above……..

<u>Sections (4): Personal protective equipment (PPE) attitude and protective measures:</u>

1) Do you use any of the following PPE (pictures of PPE were provided in the original questionnaire):

1- Facial Shield

2- Respirator

3- Eye Goggle

4- Gown (full sleeve-apron)

5- Rubber/Nitrile gloves

6- Rubber boots

7- Any other PPE? mention it……

2) Do you use it (PPE) along the whole duration of work?

- Yes
- No

Other protective measures:

1) Do you keep washing your hands regularly after work?

- Always
- Most of the time
- Sometimes
- Rarely
- Never

2) Do you keep rinsing your body regularly after work?

- Always
- Most of the time
- Sometimes
- Rarely
- Never

3) Do you keep washing the PPE after work?

- Always
- Most of the time
- Sometimes

- Rarely

- Never

4) Do you keep mercury away from food?

- Always

- Most of the time

- Sometimes

- Rarely

- Never

5) How do you dispose mercury &cyanide waste?

- Down the sink

- Dispose in river

- Combine with other waste

- Other way

6) What is the distance from work where mercury amalgam from nearest villagers?

- Less than or equal to 50 meters.

- More than 50 and less than or equal to 200 meters

- More than 200 and less than or equal to 500 meters

- More than 500 meters and less than or equal to 1 kilometer

- More than 1 and less than or equal to 3 kilometers

- More than 3 and less than or equal to 5 kilometers

- More than 5 and less than or equal to 10 kilometers

- More than 10 kilometers

Section (5): Knowledge about mercury and cyanide toxicity (options are 3; Yes, No, and I don't know except for question 14)

1- Mercury is dangerous to human beings

2- Cyanide gas affect human health & can rapidly kill human

3- Do you Prefer a safer alternative for mercury & cyanide salts

4- Mercury vapor is seen by naked eye

5- Cyanide gas is seen by naked eye

6- Mercury vapors is more toxic than ingested metallic mercury

7- Inhalation of Cyanide gas in large doses can kill in 6-8 hours by Asphyxia

8- Smoking cigarettes is a source of Cyanide

9- Mercury affect CNS (motor & coordination) on long term exposure

10- Mercury affect renal function on long term toxicity

11- Mercury affect lungs on short term

12- Mercury toxicity can cause congenital anomalies like deafness, blindness & microcephaly

13- Mercury is a cause of early miscarriage

14- Mercury toxicity is treated by:

Dialysis

Chelating agent

Activated Charcoal

Don't know

15- Cyanide toxicity is treated by IV Hydroxy-cobalamin

16- Mercury environmental hazards on water sources (rivers-wells), Domestic animals, & livestock especially young animals

17- Mercury disposed in water sources accumulate in fish, precipitated in fish is a source of highly toxic mercury
